# Impact of the pretreatment prognostic nutritional index on the survival after first‐line immunotherapy in non‐small‐cell lung cancer patients

**DOI:** 10.1002/cam4.6110

**Published:** 2023-05-21

**Authors:** Yuka Oku, Gouji Toyokawa, Sho Wakasu, Fumihiko Kinoshita, Shinkichi Takamori, Kenji Watanabe, Naoki Haratake, Taichi Nagano, Keisuke Kosai, Kazuki Takada, Airi Fujimoto, Kodo Higashijima, Yoshimasa Shiraishi, Kentaro Tanaka, Hiroaki Takeoka, Masaki Okamoto, Takanori Yamashita, Mototsugu Shimokawa, Fumihiro Shoji, Koji Yamazaki, Tatsuro Okamoto, Takashi Seto, Hitoshi Ueda, Sadanori Takeo, Naoki Nakashima, Isamu Okamoto, Tomoyoshi Takenaka, Tomoharu Yoshizumi

**Affiliations:** ^1^ Department of Surgery and Science, Graduate School of Medical Sciences Kyushu University Fukuoka Japan; ^2^ Department of Thoracic Surgery Clinical Research Institute, National Hospital Organization, Kyushu Medical Center Fukuoka Japan; ^3^ Department of Surgery National Hospital Organization Fukuoka National Hospital Fukuoka Japan; ^4^ Department of Thoracic Oncology National Hospital Organization Kyushu Cancer Center Fukuoka Japan; ^5^ Saiseikai Fukuoka General Hospital Fukuoka Japan; ^6^ Department of Pharmacy Clinical Research Institute, National Hospital Organization, Kyushu Medical Center Fukuoka Japan; ^7^ Department of Pharmacy National Hospital Organization Fukuoka National Hospital Fukuoka Japan; ^8^ Department of Respiratory Medicine, Graduate School of Medical Sciences Kyushu University Fukuoka Japan; ^9^ Department of Respiratory Medicine Clinical Research Institute, National Hospital Organization, Kyushu Medical Center Fukuoka Japan; ^10^ Medical Information Center Kyushu University Hospital Fukuoka Japan; ^11^ Department of Biostatistics, Graduate School of Medicine Yamaguchi University Yamaguchi Japan

**Keywords:** chemoimmunotherapy, first‐line immunotherapy, monotherapy, non‐small‐cell lung cancer, prognostic nutritional index

## Abstract

**Background:**

Immunotherapy has become a standard‐of‐care for patients with non‐small‐cell lung cancer (NSCLC). Although several biomarkers, such as programmed cell death‐1, have been shown to be useful in selecting patients likely to benefit from immune checkpoint inhibitors (ICIs), more useful and reliable ones should be investigated. The prognostic nutritional index (PNI) is a marker of the immune and nutritional status of the host, and is derived from serum albumin level and peripheral lymphocyte count. Although several groups reported its prognostic role in patients with NSCLC receiving a single ICI, there exist no reports which have demonstrated its role in the first‐line ICI combined with or without chemotherapy.

**Materials and Methods:**

Two‐hundred and eighteen patients with NSCLC were included in the current study and received pembrolizumab alone or chemoimmunotherapy as the first‐line therapy. Cutoff value of the pretreatment PNI was set as 42.17.

**Results:**

Among 218 patients, 123 (56.4%) had a high PNI (≥42.17), while 95 (43.6%) had a low PNI (<42.17). A significant association was observed between the PNI and both the progression‐free survival (PFS; hazard ratio [HR] =  0.67, 95% confidence interval [CI]: 0.51–0.88, *p* =  0.0021) and overall survival (OS; HR = 0.46, 95% CI: 0.32–0.67, *p* < 0.0001) in the entire population, respectively. The multivariate analysis identified the pretreatment PNI as an independent prognosticator for the PFS (*p* =  0.0011) and OS (*p*  < 0.0001), and in patients receiving either pembrolizumab alone or chemoimmunotherapy, the pretreatment PNI remained an independent prognostic factor for the OS (*p* = 0.0270 and 0.0006, respectively).

**Conclusion:**

The PNI might help clinicians appropriately identifying patients with better treatment outcomes when receiving first‐line ICI therapy.

## 1 INTRODUCTION

Lung cancer remains one of the most common causes of cancer death worldwide, and non‐small‐cell lung cancer (NSCLC) represents about 85% of lung cancer cases.^1^ Several treatments have led to the successful management of advanced or metastatic NSCLC over the past few decades. Following the emergence of molecular‐targeted agents, such as tyrosine kinase inhibitors, newly developed agents of immune checkpoint inhibitors (ICIs), programmed cell death‐ligand 1 (PD‐L1), and programmed cell death‐1 (PD‐1) inhibitors have rapidly become the standard‐of‐care for NSCLC.

However, the objective response rate (ORR) of the first‐line treatment achieved by anti‐PD‐L1/PD‐1 antibodies range from 31.1% to 46.1% despite their innovative therapeutic strategy.^2^ In the clinical setting and clinical trials, the PD‐L1 tumor proportion score (TPS) and tumor mutation burden (TMB) are adopted to predict the response to ICIs; however, these factors have been gradually proven not to be definitive biomarkers. Therefore, useful, reproducible, inexpensive biomarkers, which help identifying patients likely to be successfully treated with ICIs, have been sought. Our group previously identified several blood‐based biomarkers predictive of the antitumor efficacy, including survival, in NSCLC patients receiving ICIs.^3^, ^4^

Recently, several reports have suggested that host factors, such as the nutritional status, are as essential as the tumor biology to the clinical outcomes, including the physical condition, surgical complications, therapeutic response, cancer progression, and prognosis.^5^, ^6^ The prognostic nutritional index (PNI), an immune‐nutritional score, was originally reported in 1980^7^ and is derived from the albumin level and the count of peripheral lymphocyte, suggesting that the PNI reflects the immune and nutritional status of the host. In addition to its ability to predict the survival in the resected NSCLC, the pretreatment PNI was reportedly useful for the prediction of the ICI response in NSCLC patients.^3^ However, no reports have described its significance concerning the first‐line use of ICIs.

We therefore investigated the clinical role of the pretreatment PNI in patients with advanced or postoperative recurrent NSCLC receiving first‐line immunotherapy at four institutions.

## 2 MATERIALS AND METHODS

### 2.1 Patients

We retrospectively identified 234 NSCLC patients without multiple primary lesions or malignancies of other organs who had advanced or recurrent disease and had been treated with first‐line pembrolizumab monotherapy and chemoimmunotherapy from January 2016 to March 2021 at Kyusyu University Hospital, Kyusyu Medical Center, Fukuoka National Hospital or Kyusyu Cancer Center. Thirteen patients with an Eastern Cooperative Oncology Group (ECOG) performance status (PS) of 3 or 4 and whose PNI values were unavailable were excluded. Thus, a total of 218 patients were finally included in this retrospective study. The patients intravenously received pembrolizumab monotherapy or platinum doublet chemotherapy plus ICI, including pembrolizumab and atezolizumab, every 3 weeks (Table S2).

Ethical approval from the appropriate institutional review boards (IRB) was obtained for this study: Kyusyu University Hospital (IRB No. 2021‐103), Kyusyu Medical Center (IRB No. 21C083), Fukuoka National Hospital (IRB No. F3‐8) and Kyusyu Cancer Center (IRB No. 2021‐21).

### 2.2 Patients' clinicopathological features

All clinicopathological information were harvested from medical records. The age, sex, smoking history, body mass index (BMI), ECOG PS, regimen, values of serum albumin, and peripheral lymphocyte count, histology and the PD‐L1 TPS were determined before patients' treatment. A 22C3 pharmDx assay (Dako North America, Inc., Agilent/Dako) was used for the immunohistochemical analysis for PD‐L1, and its membranous expression in tumor cells was evaluated by TPS. Periodic radiologic tests, such as computed tomography (CT), magnetic resonance imaging, and positron emission tomography/CT, were used to assess changes in the tumor size, and the response rate was determined according to the Response Evaluation Criteria in Solid Tumors version 1.1.^8^ Immune‐related adverse events (irAEs) were determined by the Common Terminology Criteria for Adverse Events, version 5.0, through the period of observation. The median follow‐up period was 14.4 (range: 0.5–52.0) months after the initial day of the first‐line therapy.

### 2.3 The pretreatment PNI and its cutoff value

The blood tests were performed within 1 month before the start of first‐line pembrolizumab alone or ICIs with chemotherapy, and the PNI values prior to the treatment were obtained by 10 × serum albumin concentration (g/dL) + 0.005 × peripheral lymphocyte count (/mm^3^). In consideration of the median follow‐up period (14.4 months), the cutoff value was defined by time‐dependent receiver operating characteristic (ROC) curve analyses at 12 months after the administration of first‐line pembrolizumab alone or combination therapy of ICIs with chemotherapy for the prediction of the overall survival (OS); the value was 42.17 (area under the ROC curve: 0.72).^9^ Determination of the cutoff value led to the stratification of the patients included into two groups: high or low PNI.

### 2.4 Statistical analyses

Associations between categorical variables, such as patient characteristics, and the PNI or response rate were analyzed using Pearson's *χ* ^2^ test and Fisher's exact test. The progression‐free survival (PFS) was defined as the period from the initiation of the therapy to clinical or radiographic progressive disease (PD), and the OS was defined as the time from the start of the therapy until the day of the last follow‐up or death from any cause. The survival curves constructed by the Kaplan–Meier method were compared between the high‐ and low‐PNI groups using a log‐rank test and hazard ratios (HRs). Cox proportional hazard model were conducted to estimate HR, and performed to evaluate risk factor for the PFS and OS with the backward elimination procedure in which a *p* value‐based elimination method was employed. The time‐dependent ROC curve to define the cutoff value of PNI was described using the R package (https://cran.r‐project.org/web/packages/timeROC/index.html). Any items that had not been measured, collected or written in the medical record were considered as missing data. In the current study, the missing data were not complemented using the estimated or calculated values. All statistical analyses except for the description of the time‐dependent ROC curve were performed using the JMP® ver. 14.0 and SAS ver. 9.4 (SAS Institute Inc.).

## 3 RESULTS

### 3.1 Patient characteristics and their association with PNI

The clinicopathological characteristics of the 218 patients are presented in Table S1. The median age of all patients was 69 years old with a range of 36 –85 years old. One hundred and sixty‐five patients (75.7%) were male, and a smoking history was present in 185 patients (84.9%). Forty‐three patients (19.7%) had a BMI less than 18.5. One hundred and seventy patients (78.0%) showed a PD‐L1 (22C3) expression of >1%, and pembrolizumab monotherapy and chemoimmunotherapy as the first‐line setting were administered to 91 patients (41.7%) and 127 patients (58.3%), respectively. The PS distribution was as follows: 0 (49.1%), 1 (43.1%) and 2 (6.0%). During the observation period, irAEs were observed in 101 patients (46.3%).

**FIGURE 1:**
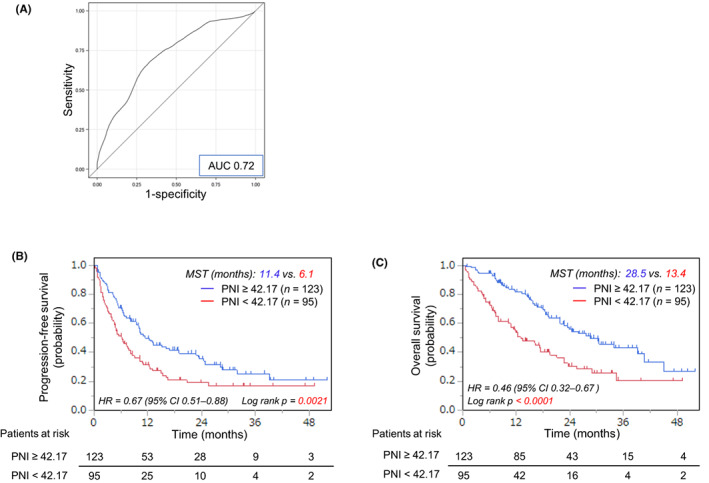
(A) Time‐dependent receiver operating characteristic curve to define the cutoff point of the prognostic nutritional index (PNI); Kaplan–Meier curves of (B) the progression‐free survival and (C) the overall survival for 218 non‐small‐cell lung cancer patients treated with first‐line pembrolizumab monotherapy or chemoimmunotherapy according to the cutoff point of the pretreatment PNI; AUC, area under the curve; CI, confidence interval; HR, hazard ratio; MST, median survival time; PNI, prognostic nutritional index.

The median pretreatment PNI was 44.0 (range: 21.9–65.2). The cutoff value was determined as 42.17, according to the time‐dependent ROC curve to predict the OS at 12 months. This value separated the 218 patients into a high‐PNI group of 123 (56.4%) and low‐PNI group of 95 (43.6%) (Figure 1A). As shown in Table 1, a statistically significant difference was observed between the groups in the ECOG PS and pretreatment PNI value (*p* < 0.0001).

**TABLE 1:** Clinicopathological characteristics for each PNI group and comparison between groups (*N* = 218).

		PNI group		
		High^a^ (*N* = 123)	Low^b^ (*N* = 95)	
Characteristics	*N*	*N* (%)	*N* (%)	*p* value
Age, years				
<70	120	67 (54.5%)	53 (55.8%)	0.8462
≥70	98	56 (45.5%)	42 (44.2%)	
Sex				
Female	53	32 (26.0%)	21 (22.1%)	0.5045
Male	165	91 (74.0%)	74 (77.9%)	
Smoking^c^				
Never	32	23 (18.7%)	9 (9.5%)	0.0532
Current or ex	185	99 (80.5%)	86 (90.5%)	
BMI				
<18.5	43	19 (15.4%)	24 (25.3%)	0.0709
≥18.5	175	104 (84.6%)	71 (74.7%)	
PS^c^				
0	107	78 (63.4%)	29 (30.5%)	<0.0001
1	94	40 (32.5%)	54 (56.8%)	
2	13	2 (1.6%)	11 (11.6%)	
Histology^c^				
Sq	51	27 (22.0%)	24 (25.3%)	0.6125
Non‐Sq	165	94 (76.4%)	71 (74.7%)	
PD‐L1 (22C3) TPS^c^				
<1%	36	26 (21.1%)	10 (10.8%)	0.0613
≥1%	170	94 (76.4%)	76 (80.0%)	
Therapy				
ICI	91	52 (42.3%)	39 (41.1%)	0.8558
ICI + Chemo	127	71 (57.7%)	56 (58.9%)	
irAEs				
Present	101	58 (47.2%)	43 (45.3%)	0.7813
Absent	117	65 (52.8%)	52 (54.7%)	

Abbreviations: BMI, body mass index; Chemo, chemotherapy; Current, current smoker; ECOG, Eastern Cooperative Oncology Group; ex, ex‐smoker; ICI, immune checkpoint inhibitor; irAEs, immune‐related adverse events; Non‐Sq, non‐squamous cell carcinoma; PD‐L1, programmed cell death‐ligand 1; PNI, prognostic nutritional index; PS, performance status; Sq, squamous cell carcinoma; TPS, tumor proportion score.

^a^ Value more than 42.17.

^b^ Value less than 42.17.

^c^ Cases for which data were available; smoking (*N* = 217), ECOG PS (*N* = 214), histology (*N* = 216) and PD‐L1 (22C3) TPS (*N* = 206).

### 3.2 The PFS and OS according to the pretreatment PNI

The Kaplan–Meier curves of the PFS and OS for the 218 patients receiving pembrolizumab monotherapy or chemoimmunotherapy as the first‐line therapy are shown in Figure S1A,B, and there were no significant differences in the PFS or OS between monotherapy and ICI plus chemotherapy (Figure S1C,D). Figure 1 shows the survival curves for the high‐ and low‐PNI groups, and the low‐PNI group exhibited a significantly poorer PFS than the high‐PNI group (median PFS: high‐PNI vs. low‐PNI = 11.4 vs. 6.1 months, *p* = 0.0021 [Figure 1B] and median OS: high‐PNI vs. low‐PNI = 28.5 vs. 13.4 months, *p* < 0.0001 [Figure 1C]). In addition, when the patients were stratified by PD‐L1 expression, PNI still remained a predictor for both PFS and OS in patients with PD‐L1 ≥50% (Figures S2A,B) and PD‐L1 <50% (Figures S2C,D).

### 3.3 Prognostic factors in the first‐line setting for pembrolizumab monotherapy and chemoimmunotherapy

We investigated the prognosticators for the PFS and OS in all the patients treated with pembrolizumab monotherapy or chemoimmunotherapy using univariate and multivariate analyses (Table 2). Multivariate analyses demonstrated that PS (*p* = 0.0015) and the PNI value (*p* = 0.0011) significantly affected the PFS with HRs of 3.53 (95% confidence interval [CI]: 1.89–6.02) and 1.73 (95% CI: 1.24–2.39) for the worse ECOG PS and a low PNI, respectively. Regarding the OS, ECOG PS (*p* = 0.0007) and the PNI (*p* < 0.0001) were independent predictors in the multivariate analysis; the HRs were 3.38 (95% CI: 1.75–5.96) for an ECOG PS of 2 and 2.17 (95% CI: 1.50–3.16) for a low PNI, respectively. We also performed the analysis with the cutoff value of PD‐L1 ≥50%; however, PNI still remained predictive of both PFS and OS (data not shown).

**TABLE 2:** Multivariate analyses of clinicopathological factors associated with the PFS and OS in 218 non‐small‐cell lung cancer patients treated with first‐line pembrolizumab monotherapy or chemoimmunotherapy.

		PFS	Multivariate analysis		OS	Multivariate analysis	
Characteristics		HR	95% CI	*p* value	HR	95% CI	*p* value
Age, years	≥70	0.98	0.69–1.39	0.9161	1.21	0.80–1.81	0.3646
Sex	Male	1.52	0.94–2.55	0.0872	1.59	1.00–2.66	0.0515
Smoking	Current or ex	0.81	0.53–1.29	0.3596	0.57	0.31–1.11	0.0945
BMI	<18.5	1.07	0.67–1.64	0.7757	1.27	0.77–2.03	0.3394
PS	≥2	3.53	1.89–6.02	0.0015	3.38	1.75–5.96	0.0007
Histology	Sq	1.17	0.77–1.74	0.4525	1.33	0.84–2.06	0.2186
PD‐L1 (22C3) TPS	<1%	1.47	0.91–2.29	0.1157	1.74	0.98–2.94	0.0593
Therapy	ICI	1.18	0.81–1.72	0.3881	1.24	0.80–1.94	0.3424
PNI	<42.17	1.73	1.24–2.39	0.0011	2.17	1.50–3.16	<0.0001

Abbreviations: BMI, body mass index; CI, confidence interval; Current, current smoker; ECOG, Eastern Cooperative Oncology Group; ex, ex‐smoker; HR, hazard ratio; ICI, immune checkpoint inhibitor; OS, overall survival; PD‐L1, programmed cell death‐ligand 1; PFS, progression‐free survival; PNI, prognostic nutritional index; PS, performance status; Sq, squamous cell carcinoma; TPS, tumor proportion score.

### 3.4 The PFS and OS after the start of initial therapy with pembrolizumab alone or immunotherapy combined with chemotherapy according to the PNI

**FIGURE 2:**
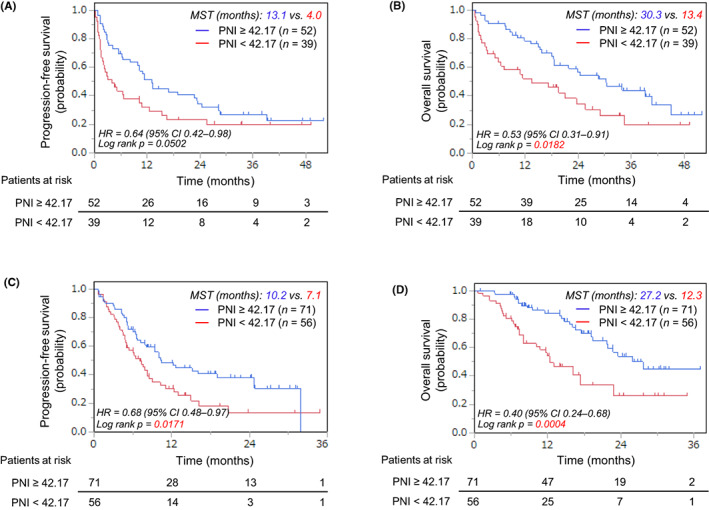
Kaplan–Meier curves of (A, C) the progression‐free survival and (B, D) the overall survival for patients treated with first‐line (A, B) immune checkpoint inhibitor monotherapy and (C, D) immune checkpoint inhibitor combination therapy according to cutoff point of pretreatment prognostic nutritional index level; CI, confidence interval; HR, hazard ratio; MST, median survival time; PNI, prognostic nutritional index.

Figure 2A,B show the PFS and OS in the 91 patients who received monotherapy, while Figure 2C,D show the PFS and OS in the 127 patients who received chemoimmunotherapy by Kaplan–Meier analyses. The significances were verified using the log‐rank test. The PFS did not significantly differ between the low‐ and high‐PNI patients who received monotherapy but differed in those who received chemoimmunotherapy (*p* = 0.0502 and 0.0171, respectively [Figure 2A,C]). There were significant differences in the OS between the low‐ and high‐PNI groups (*p* = 0.0182 and 0.0004, respectively [Figure 2B,D]).

Finally, we assessed the relationship between the survival and each categorical value by multivariate analyses. A low PNI was shown to be a prognosticator for the OS by the multivariate analysis (HR = 1.86, *p* = 0.0270; HR = 2.49, *p* = 0.0006 in the monotherapy and chemoimmunotherapy populations, respectively [Tables 3 and 4]). For the PFS, a low PNI was a predictor only in the chemoimmunotherapy (HR = 1.68, *p* = 0.0190 [Table 4]). The cutoff value of PD‐L1 ≥50% did not predict the PFS and OS (data not shown).

**TABLE 3:** Multivariate analyses of clinicopathological factors associated with the PFS and OS in 91 non‐small‐cell lung cancer patients treated with first‐line pembrolizumab monotherapy.

		PFS	Multivariate analysis		OS	Multivariate analysis	
Characteristics		HR	95% CI	*p* value	HR	95% CI	*p* value
Age, years	≥70	1.46	0.76–3.03	0.2687	1.80	1.01–3.20	0.0470
Sex	Male	1.33	0.71–2.47	0.3713	1.19	0.61–2.57	0.6227
Smoking	Current or ex	0.71	0.39–1.45	0.3361	0.69	0.29–1.81	0.4342
BMI	<18.5	1.14	0.56–2.16	0.7078	1.16	0.55–2.27	0.6867
ECOG PS	≥2	3.20	1.32–6.64	0.0129	4.07	1.66–8.60	0.0038
Histology	Sq	1.16	0.63–2.08	0.6188	1.34	0.72–2.39	0.3400
PNI	<42.17	1.62	0.99–2.63	0.0554	1.86	1.07–3.19	0.0270

Abbreviations: BMI, body mass index; CI, confidence interval; Current, current smoker; ECOG, Eastern Cooperative Oncology Group; ex, ex‐smoker; HR, hazard ratio; ICI, immune checkpoint inhibitor; OS, overall survival; PFS, progression‐free survival; PNI, prognostic nutritional index; PS, performance status; Sq, squamous cell carcinoma.

**TABLE 4:** Multivariate analyses of clinicopathological factors associated with the PFS and OS in 127 non‐small‐cell lung cancer patients treated with first‐line chemoimmunotherapy.

		PFS	Multivariate analysis		OS	Multivariate analysis	
Characteristics		HR	95% CI	*p* value	HR	95% CI	*p* value
Age, years	≥70	0.72	0.45–1.14	0.1632	0.91	0.51–1.60	0.7468
Sex	Male	2.13	1.00–5.08	0.0512	1.96	1.00–4.16	0.0520
Smoking	Current or ex	0.69	0.40–1.29	0.2367	0.38	0.16–0.96	0.0411
BMI	<18.5	1.14	0.60–2.06	0.6702	1.27	0.63–2.44	0.4943
ECOG PS	≥2	1.94	0.53–5.64	0.2912	1.40	0.30–4.60	0.6327
Histology	Sq	1.11	0.58–1.98	0.7381	1.19	0.56–2.35	0.6278
PD‐L1 (22C3) TPS	<1%	1.54	0.92–2.50	0.0955	1.86	1.01–3.30	0.0452
PNI	<42.17	1.68	1.09–2.60	0.0190	2.49	1.48–4.25	0.0006

Abbreviations: BMI, body mass index; CI, confidence interval; Current, current smoker; ECOG, Eastern Cooperative Oncology Group; ex, ex‐smoker; HR, hazard ratio; ICI, immune checkpoint inhibitor; irAEs, OS, overall survival; PD‐L1, programmed cell death‐ligand 1; PFS, progression‐free survival; PNI, prognostic nutritional index; PS, performance status; Sq, squamous cell carcinoma; TPS, tumor proportion score.

### 3.5 Associations between the response rate and clinicopathological characteristics

Subsequently, the associations between the response rate and pretreatment PNI were assessed in patients by treatment type. Among the 197 patients whose response data were available, 80 were treated with pembrolizumab monotherapy, while 117 were with chemoimmunotherapy. The former included 42 patients (52.5%) with an ORR and 38 patients (47.5%) with stable disease (SD) or PD. The latter did 70 patients (59.8%) with ORR and 47 patients (40.2%) in SD or PD. Table S3 and Figure S3 showed a non‐significant trend for the high‐PNI group to have a higher ORR than the frequency of stable or progressive disease in the monotherapy group.

## 4 DISCUSSION

The present study is the first to show that, in NSCLCs treated with first‐line ICI‐based therapy, the pretreatment PNI was an independent prognostic factor; patients with a high PNI had a significantly better PFS and OS than those with a low PNI. In addition, the pretreatment PNI showed potential to predict the OS in both pembrolizumab monotherapy and chemoimmunotherapy patients.

Recently, ICIs have been recognized as particularly promising treatment choices because of their additive effects with chemotherapy, even in the absence of PD‐L1 expression.^10^, ^11^ This makes it more imperative than ever to identify appropriate biomarkers, since which patients can truly benefit from ICIs is not sufficiently clear. Blood‐based biomarkers and radiological findings have been identified to potentially predict the survival in NSCLC patients receiving first‐line ICI‐based therapy.^12^, ^13^, ^14^ Regarding first‐line chemoimmunotherapy, a prospective cohort by Charu et al. demonstrated that a plasma‐based TMB of >16 mutations per megabase was associated with an improved PFS.^15^

A number of reports have additionally suggested that a host's immunonutritional status may also be a good biomarker which helps predicting the outcomes of ICI‐based treatments. Such an immunonutritional status can be estimated in an inexpensive and simple manner using blood tests. We reported the clinical utility of the PNI in ICI monotherapy beyond first‐line treatment.^3^, ^16^, ^17^ The PNI was also reported to predict postoperative complications, the disease‐free survival, the PFS, the OS and responsiveness to chemotherapy in several types of malignancies.^3^, ^18^, ^19^, ^20^, ^21^, ^22^, ^23^, ^24^ Thus, the PNI might be prognostic regardless of types of treatment; however, this is a first report to reveal the PNI to be a useful prognostic indicator in NSCLC who received first‐line pembrolizumab monotherapy or chemoimmunotherapy.

It is widely acknowledged that the serum albumin level reflects the nutritional status of cancer patients, and it is known negatively correlate with systemic inflammatory response.^25^ In addition, the peripheral absolute lymphocyte count reflects the status of the acquired immune system, including chronic inflammation, and its positive association with tumor‐infiltrating lymphocytes was also reported.^26^, ^27^ Thus, the PNI, which is the additive value of the albumin level and lymphocyte count, might predict the response to ICI, since the PNI may reflect the local microenvironment relevant for anti‐tumor immunity and the nutritional change of the host.

This study set the cutoff value of the PNI at 42.17 based on the time‐dependent ROC curve at 12 months, given the median observation period (14.4 months). This cutoff value did not result in any specific bias in the patients' background factors, except for the PS, which seemed to be related to the host's nutritional status (Table 1). In a series of reports investigating the significance of the PNI for predicting the treatment outcomes of lung cancer patients undergoing surgery or receiving chemotherapy and ICIs, the cutoff value ranged from 35 to 50, respectively.^3^, ^27^, ^28^, ^29^, ^30^ Thus, the cutoff value of PNI differs among studies, and further investigations will be needed to identify the optimum cutoff value unique to each treatment setting.

This study is associated with limitations due to its retrospective nature. Several biases, such as the exclusion of patients with a PS of 3 and 4, certain regimens, histological types and the lack of PNI data in several patients, might have influenced the results shown in this study. In addition, because of the limited number of the patients included and the heterogeneity of treatment groups, it was difficult to set up a validation cohort. Nonetheless, this is the largest multi‐center study ever discussing the relationship between the PNI and patients' outcome after immunotherapy with or without chemotherapy in the first‐line setting. Furthermore, the survival rates were almost similar to those reported in the previous trials, suggesting the results obtained were credible.^31^, ^32^, ^33^ An ongoing multicenter prospective study is expected to resolve the abovementioned bias.^34^

Finally, the pretreatment PNI was identified to independently predict patients' survival when administering first‐line treatment with pembrolizumab alone or chemoimmunotherapy, suggesting that PNI might be useful as a simple, inexpensive, and beneficial biomarker in the clinical setting.

## AUTHOR CONTRIBUTIONS

**Yuka Oku:** Conceptualization (equal); methodology (equal); writing—original draft (equal). **Gouji Toyokawa:** Conceptualization (equal); investigation (equal); project administration (equal); writing—review and editing (equal). **Sho Wakasu:** Data curation (supporting). **Fumihiko Kinoshita:** Data curation (supporting). **Shinkichi Takamori:** Data curation (supporting). **Kenji Watanabe:** Data curation (supporting). **Naoki Haratake:** Data curation (supporting); writing—review and editing (supporting). **Taichi Nagano:** Data curation (supporting). **Keisuke Kosai:** Data curation (supporting). **Kazuki Takada:** Data curation (supporting). **Airi Fujimoto:** Data curation (supporting). **Kodo Higashijima:** Data curation (supporting). **Yoshimasa Shiraishi:** Data curation (supporting). **Kentaro Tanaka:** Data curation (supporting). **Hiroaki Takeoka:** Data curation (supporting). **Masaki Okamoto:** Data curation (supporting). **Takanori Yamashita:** Data curation (supporting). **Mototsugu Shimokawa:** Formal analysis (equal); methodology (equal). **Fumihiro Shoji:** Supervision (supporting). **Koji Yamazaki:** Supervision (equal). **Tatsuro Okamoto:** Supervision (equal). **Takashi Seto:** Supervision (equal). **Hitoshi Ueda:** Supervision (equal). **Sadanori Takeo:** Supervision (equal). **Naoki Nakashima:** Supervision (equal). **Isamu Okamoto:** Supervision (equal). **Tomoyoshi Takenaka:** Supervision (lead); writing—review and editing (lead). **Tomoharu Yoshizumi:** Supervision (lead); writing—review and editing (lead).

## CONFLICT OF INTEREST STATEMENT

The authors have a potential financial conflict of interest as follows: Fumihiro Shoji has honoraria for lectures from Chugai Pharmaceutical, Eli Lilly Japan, Ono Pharmaceutical and Taiho Pharmaceutical. Tatsuro Okamoto has received research fundings from AnHeart Therapeutics, AstraZeneca, Bristol‐Myers Squibb, Chugai Pharmaceutical, Covidien Japan, Daiichi Sankyo, Eli Lilly Japan, KM Biologics, Merck Biopharma, MSD, Nippon Boehringer Ingelheim, Nippon Kayaku, Novartis Pharma, Pfizer Japan and Taiho Pharmaceutical and has honoraria for lectures from AstraZeneca, Bristol‐Myers Squibb, Chugai Pharmaceutical, Eli Lilly Japan, Johnson & Johnson, MSD, Nippon Boehringer Ingelheim, Nippon Kayaku, Novartis Pharma, Ono Pharmaceutical, Taiho Pharmaceutical and Towa Pharmaceutical. Takashi Seto has received research fundings from Abbvie, Chugai Pharmaceutical, Daiichi Sankyo, Eli Lilly Japan, Kissei Pharmaceutical, MSD, Novartis Pharma, Pfizer Japan and Takeda Pharmaceutical and has honoraria for lectures from AstraZeneca, Bristol‐Myers Squibb, Chugai Pharmaceutical, Covidien Japan, Daiichi Sankyo, Eli Lilly Japan, Kyowa Hakko Kirin, MSD, Mochida Pharmaceutical, Nippon Boehringer Ingelheim, Novartis Pharma, Ono Pharmaceutical, Pfizer Japan, Taiho Pharmaceutical, Takeda Pharmaceutical and Towa Pharmaceutical. Isamu Okamoto has received research grants and personal fees from Chugai Pharma and MSD Oncology, outside the submitted works.

## Supporting information


Figure S1.
Figure S2.
Figure S3.

Table S1.
Table S2.
Table S3.

## Data Availability

The data that support the findings of this study are available from the corresponding author upon reasonable request.
