# Supplementary material for: Impact of the pretreatment prognostic nutritional index on the survival after first‐line immunotherapy in non‐small‐cell lung cancer patients
Source: Cancer Med. 2023 May 21;12(13):14327–36. doi: 10.1002/cam4.6110 (PMC10358235; doi:10.1002/cam4.6110)
Supplement: Supplementary file 1 — Figure S1. Figure S2. Figure S3. [file CAM4-12-14327-s002.pdf]

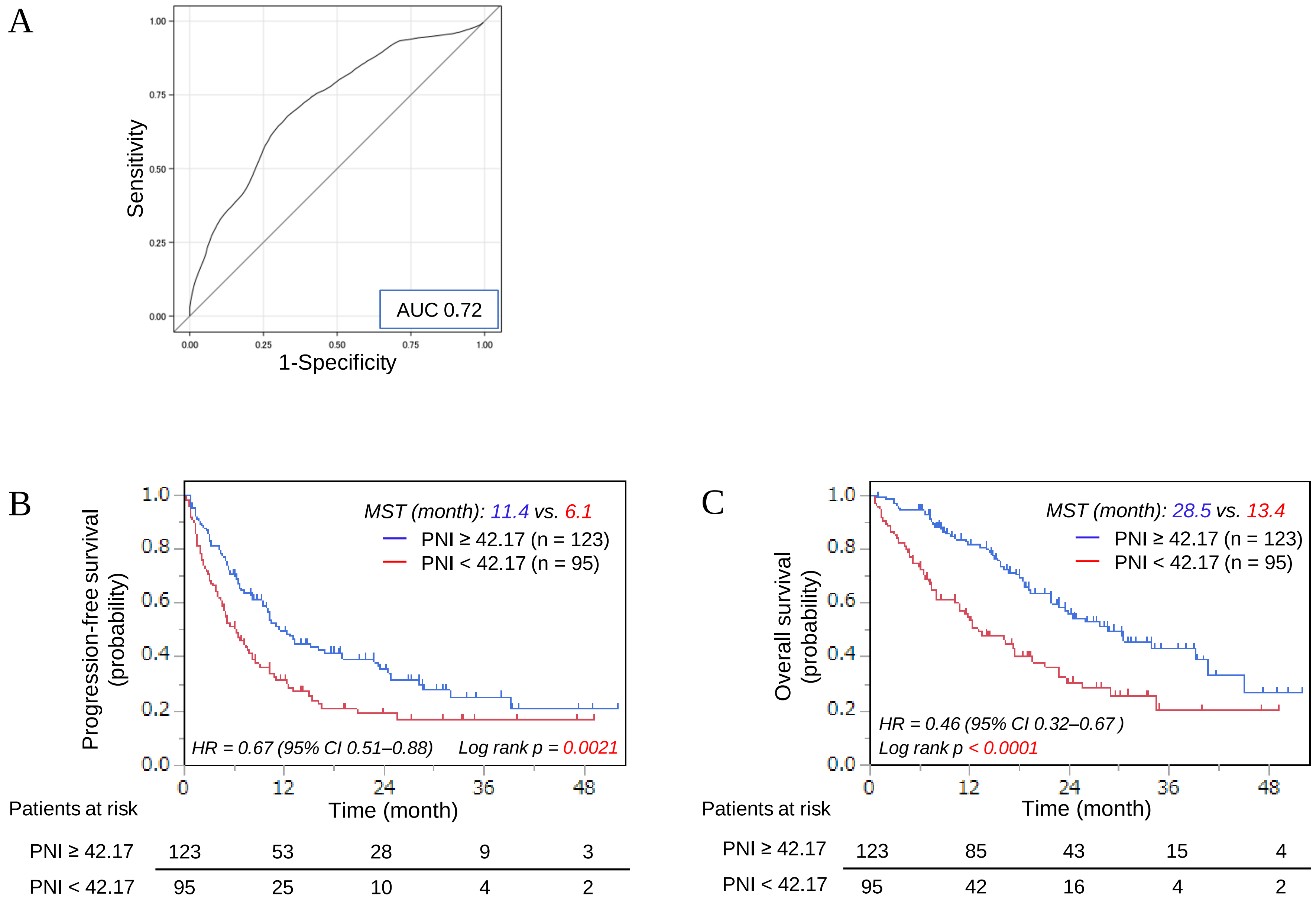

Figure 1. Oku et al.

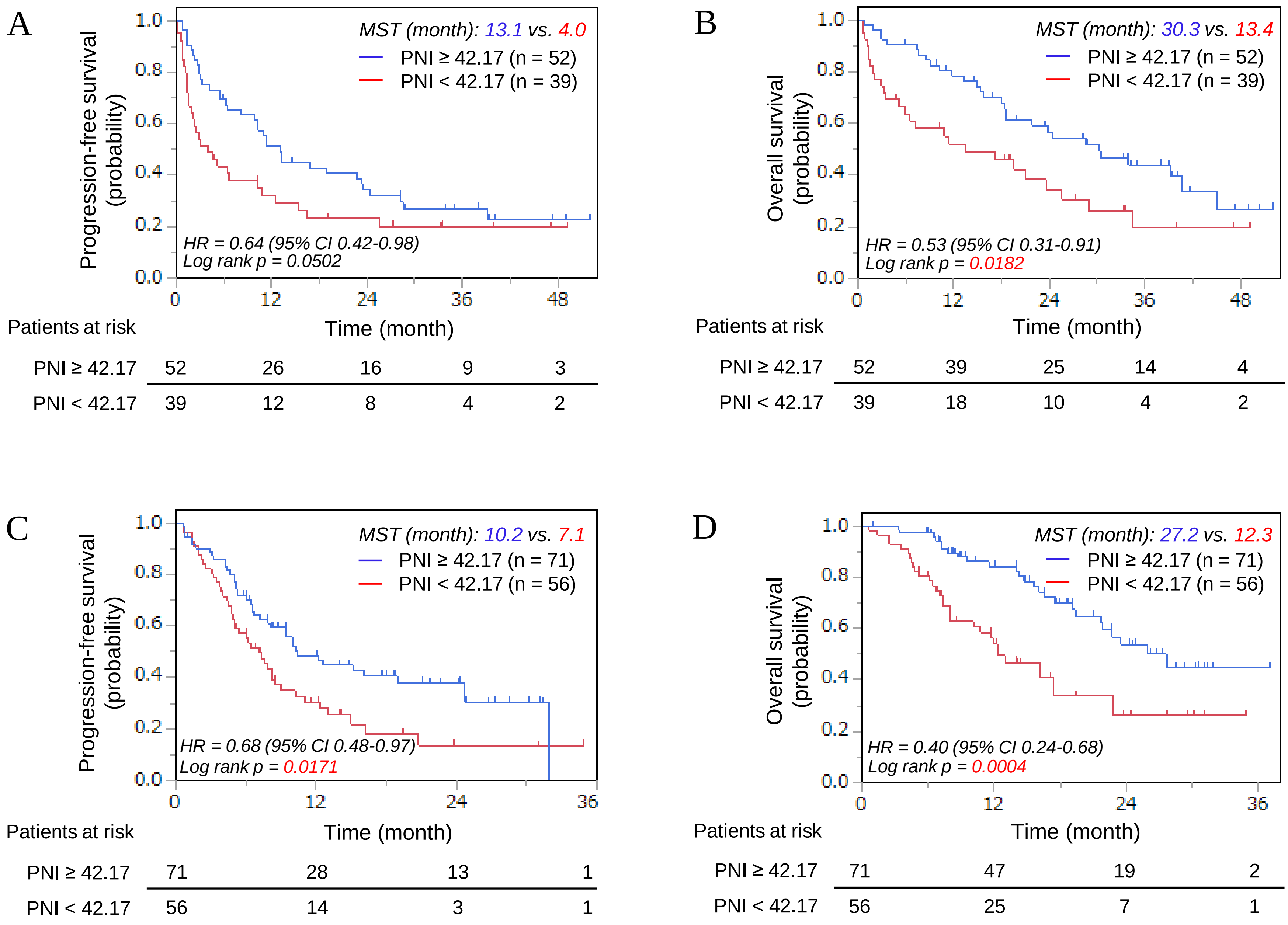

Figure 2. Oku et al.

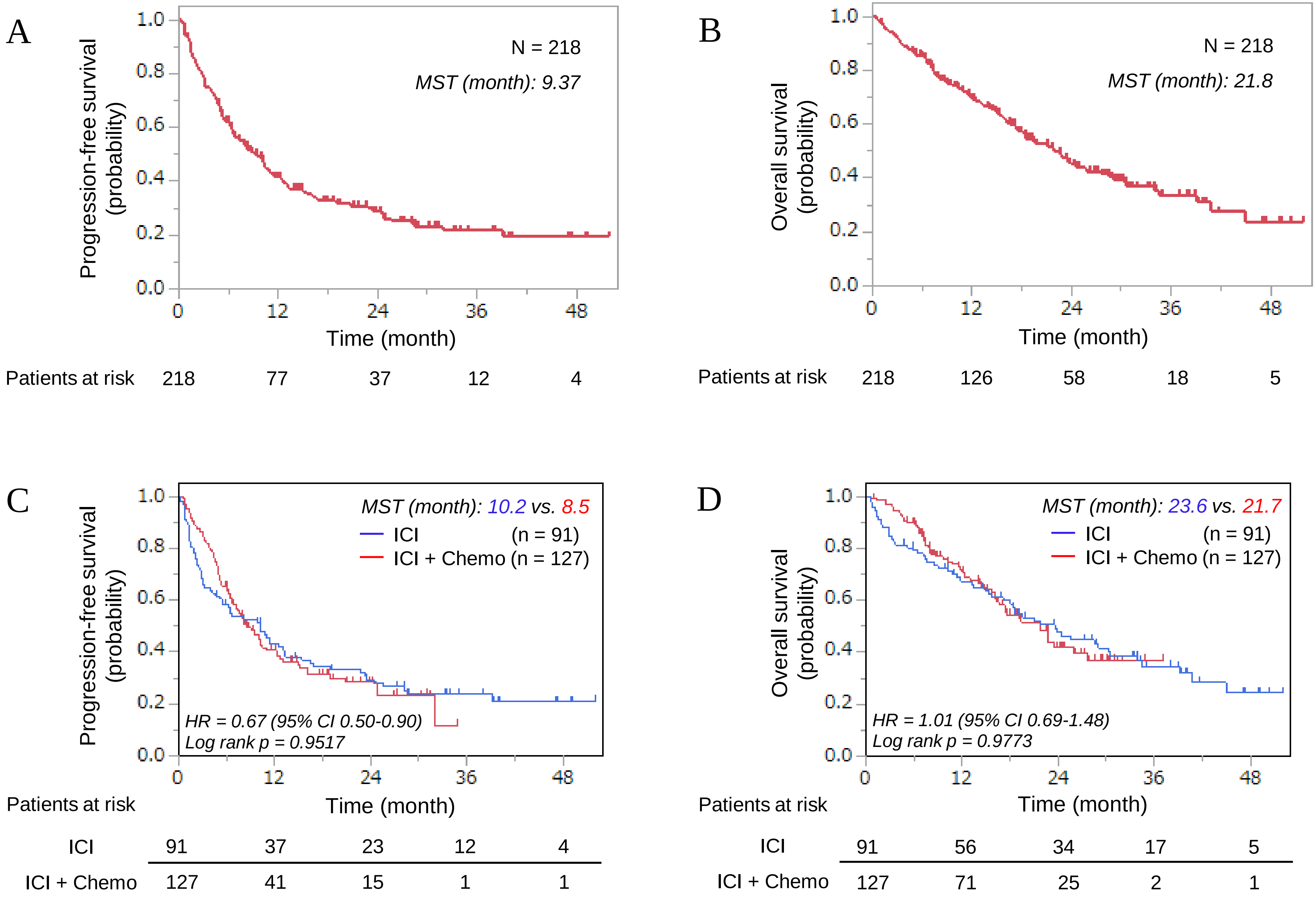

Supplementary figure 1. Oku et al.

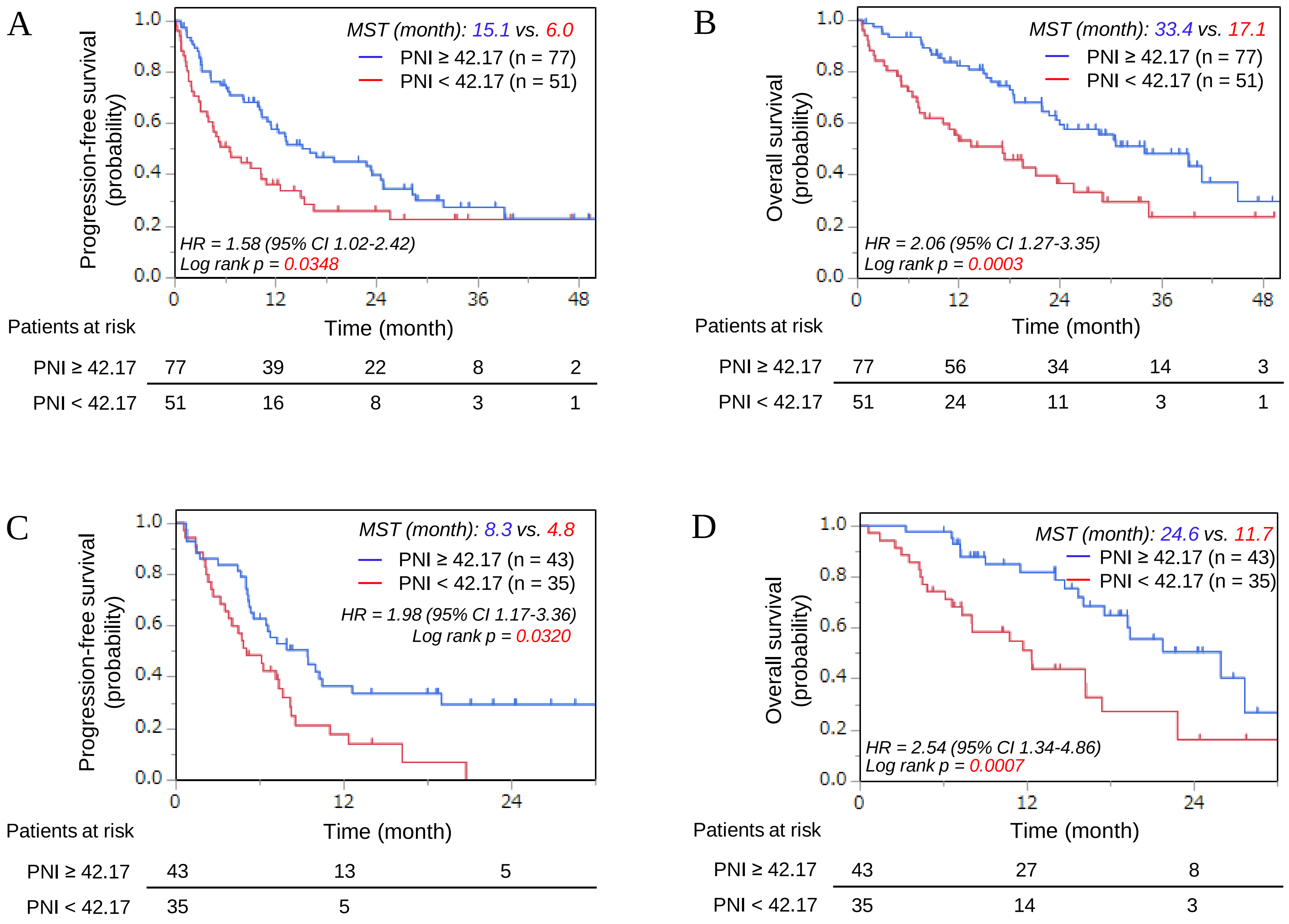

Supplementary figure 2. Oku et al.

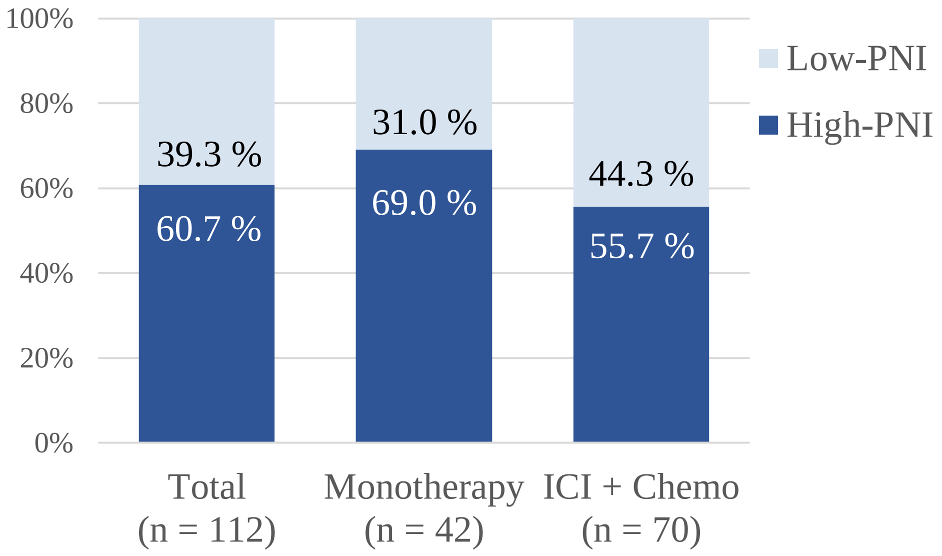

Supplementary figure 3. Oku et al.
